# Endovascular Debulking of Human Carotid Plaques by Using an Excimer Laser Combined With Balloon Angioplasty: An *ex vivo* Study

**DOI:** 10.3389/fcvm.2021.700497

**Published:** 2021-09-20

**Authors:** Kai Yang, Jinyun Tan, Ying Deng, Weihao Shi, Bo Yu

**Affiliations:** ^1^Department of Vascular Surgery, Shanghai Pudong Hospital, Fudan University Pudong Medical Center, Shanghai, China; ^2^Shanghai Key Laboratory of Vascular Lesions Regulation and Remodeling, Shanghai Pudong Hospital, Fudan University Pudong Medical Center, Shanghai, China; ^3^Department of Vascular Surgery, Huashan Hospital, Fudan University, Shanghai, China

**Keywords:** debulking device, balloon angioplasty, excimer laser, carotid atherosclerotic plaque, carotid artery revascularization

## Abstract

**Purpose:** We aimed to evaluate the safety and effectiveness of applying an excimer laser in debulking human carotid atherosclerotic plaques by investigating the distal debris, plaque luminal gain, and micromorphology of the plaque surface.

**Methods:** Eighteen plaque samples obtained from carotid endarterectomy (CEA) were randomly allocated to the excimer laser ablation (45 mJ/mm^2^, 25 Hz) alone group (group 1), balloon angioplasty (8 atm) alone group (group 2), and excimer laser ablation combined with balloon angioplasty group (group 3). Hematoxylin–eosin staining and Movat's pentachrome staining were performed on the collected particles to quantify the size and composition of the debris. The superficial micromorphological structure of the plaque lumen surface after device treatments was observed using a scanning electron microscope. Micro-CT, tissue sections, and pathological stainings were applied to the treated plaques. The plaque lumen and artery lumen were three-dimensionally reconstructed using clinical computed tomography angiography and the micro-CT images. Lumen enlargement was set as the main measurement of effectiveness.

**Results:** Group 3 produced the highest luminal gain (5.40 ± 4.51 mm^2^), while the other two groups had gains of 4.05 ± 3.20 and 3.77 ± 2.55 mm^2^. Both devices caused disruptions to the plaque lumen surface. Laser ablation exposed the fibers under the endothelium and balloon angioplasty cracked the surface. The mean amounts were 3,611 ± 1,475.4 for group 1, 2,828 ± 1,266.7 for group 2, and 4,400 ± 2,567.9 for group 3. More than 90% of the distal debris was smaller than 10 μm. Group 2 produced the most debris with Feret (maximum caliper diameter) ≥ 40 μm; group 1 had the least. There was little difference in the contents of collagen and reticular fiber in the debris in each group, but a big difference was observed in the contents of fibrin and mucin.

**Conclusion:** Excimer laser ablation could significantly increase the luminal gain of carotid plaque with high stenosis. Excimer laser combined with balloon angioplasty achieved the highest lumen enlargement. Our result also suggests that the embolic protection strategy needs to be renewed for the application of a plaque debulking device in the future.

## Introduction

Carotid artery atherosclerotic plaque and stenosis are major causes of ischemic stroke. Carotid endarterectomy (CEA) and carotid angioplasty and stenting (CAS) are two surgical treatments widely carried out to relieve carotid artery stenosis. The advantages of CEA include removing the plaque thoroughly and low 30-day stroke risk (1), but the heart failure and nerve injury rates are relatively higher. CAS is a less invasive procedure with a lower incidence of myocardial infarction and wound complications, but the stroke and death rates are higher than those of CEA (2). An ideal carotid revascularization procedure probably should combine the advantages of both techniques; that is, to not only remove the plaque under a reliable embolic protection device but also to be as mini-invasive as possible. Unfortunately, this is yet to be achieved.

An excimer laser is a pulsed gas laser with a wavelength in the ultraviolet spectrum. The exact excimer laser applied in the vascular system is generated by xenon and chlorine, with a wavelength of 308 nm. The mechanisms of excimer laser ablation to break down molecular bonds, vaporize, and remove matter include a photochemical effect, a photothermal effect, and a photomechanical effect (3). Unlike YAG (yttrium–aluminum–garnet) and CO_2_ lasers that function with a thermal effect, an excimer laser has higher security and is thermal damage-free. These characteristics make an excimer laser suitable for plaque debulking. Clinically, excimer laser ablation has been widely performed in coronary and lower extremity arteries for vessel preparation. However, an excimer laser has not yet been applied to treat carotid artery stenosis. The biggest barrier to its use in the carotid artery is the concern of complications such as perforation and distal embolization (4, 5). Although their incidence has been reported to be very low, it really could be disastrous in this region.

The present study was conducted to investigate the safety and effectiveness of applying an excimer laser in debulking human carotid atherosclerotic plaques. The parameters distal debris and luminal gain were evaluated, as well as the micromorphology of the plaque lumen surface.

## Materials and Methods

### Human Carotid Plaque Sample

**Figure 1:**
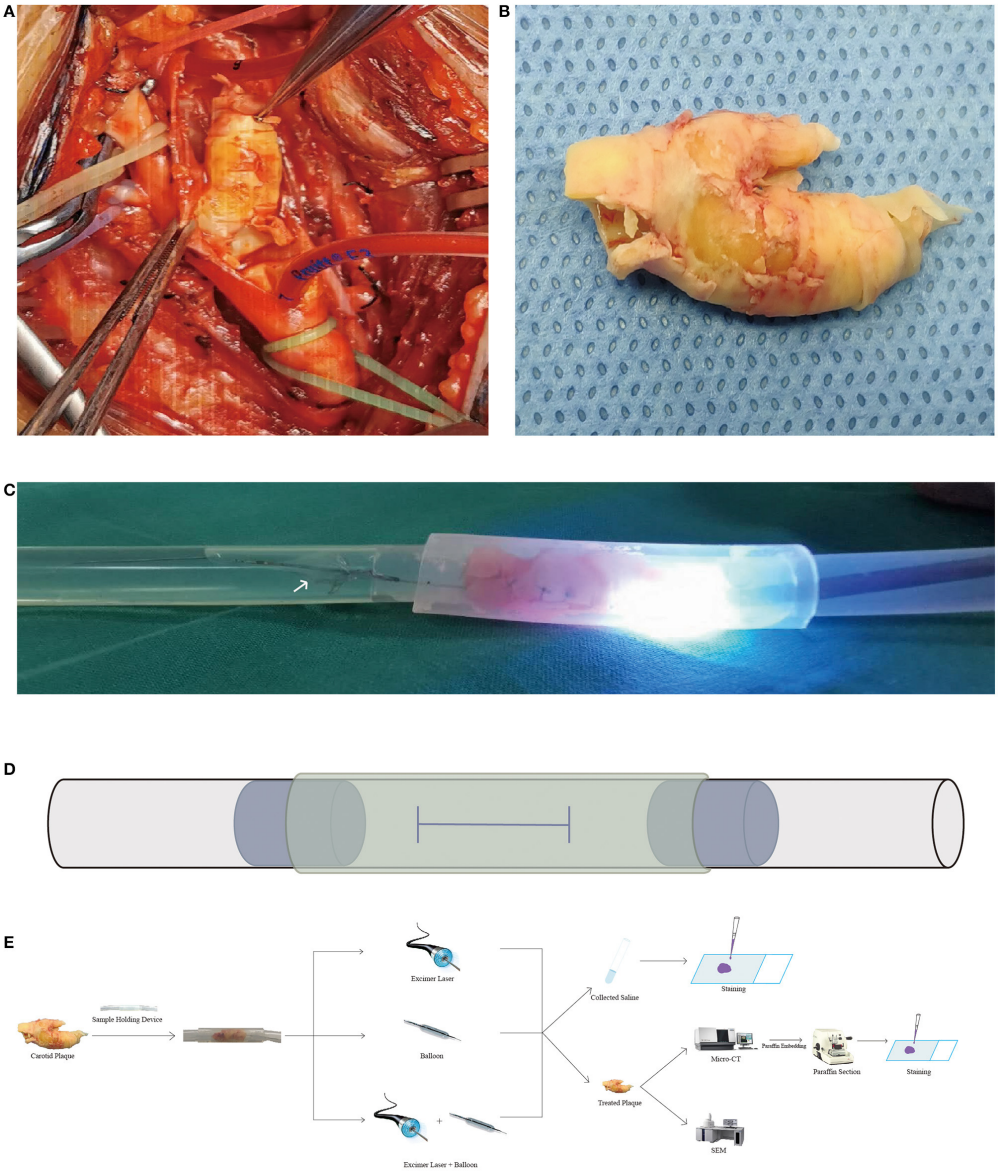
Carotid plaque images, experimental images, and flowchart. **(A,B)** Intraoperative and sample images of the carotid plaque during and after carotid endarterectomy. **(C)** Image from excimer laser ablation. A flashlight was radiated from the laser catheter. The *white arrow* indicates the distal embolic protection device. **(D)** Schematic diagram of the sample holding device. An H-shaped incision was made on the main pipe (*black solid line*) for putting the carotid plaque and two short pipes (*blue dot line*) in. Another short pipe (*yellow dashed line*) was placed over the main pipe to seal the H-shaped incision. All pipes are made of silicon and are transparent. **(E)** Flowchart of the study. Several pictures used in the flowchart were obtained from the Internet. Micro-CT, micro-computed tomography; SEM, scanning electron microscope.

All carotid plaques used in the experiments were with higher stenosis, ranging from 60 to 99%. The mean stenosis of plaques was 78.8 ± 9.2%. Human carotid plaques were obtained during CEA by intentionally using the “en bloc” technique (Figure 1A). Each plaque was immersed in a container filled with enough heparin saline. The container was then shaken gently to remove blood from the plaque. After being photographed (Figure 1B), the carotid plaques were stored in saline and transferred immediately in an icebox for further experiments. This study and the use of human carotid plaque samples were approved by the Ethics Committee of Shanghai Pudong Hospital (W2-02). The patients provided written informed consent for participation in this study. Silicon pipes of different sizes were used to construct the carotid plaque holding device. A 30-cm-long suitable silicon pipe was chosen as the main pipe based on the size of the plaque sample. An H-shaped incision was made on the main pipe. Before the plaque was gently placed into the main pipe, two short pipes were inserted into the main pipe through the incision in order to keep the plaques still. Another bigger short pipe was sleeved onto the main pipe to seal the H-shaped incision (Figure 1D).

### Study Design and Protocol

The study was carried out *ex vivo* and consisted of the following three groups: excimer laser ablation alone (group 1), balloon angioplasty alone (group 2), and excimer laser ablation combined with balloon angioplasty (group 3) (Figure 1E). Each group was randomly assigned to six carotid plaques. After the debulking procedure, micro-CT scans and histological staining were performed for all samples. In the clinical setting, the stenosis rate is the criterion for judging the success of the procedure. However, in this *ex vivo* experiment, there was a lack of suitable equipment to detect the stenosis rate after laser ablation or balloon dilation, and we failed to set the stenosis rate as the criterion. To make each group comparable, we then set the lumen area and area enlargement as the main measurements. The 308-nm XeCl excimer laser system (CVX-300 Spectranetics^®^ Philips, San Diego, CA, USA) was operated at an energy of 45 mJ/mm^2^ and a repetition rate of 25 Hz. A 0.018-in. guidewire was placed through the sample first, and then catheter advancement was performed slowly at a rate of ≥ 0.5 < 1 mm/s. When the catheter tip crossed the sample, laser irradiation was stopped and the catheter was withdrawn. The process was repeated five times based on improvements from our previous experiments. The saline infusion technique was applied at a flushing rate of 1 ml/s during catheter advancement. *Ex vivo* angioplasty was done with a 3.0-mm balloon at 8 atm. Each time the device is withdrawn, enough saline was flushed into the sample holding device to remove microdebris. All saline was collected. After the experimental procedure, the carotid samples were fixed in 4% neutral formalin solution for further experiments.

### Excimer Laser Ablation and Balloon Angioplasty Process

After the 0.018-in. guidewire crossed the carotid plaque, the laser catheter was placed against the plaque. During the excimer laser ablation procedure, a flashing light and a beeping sound were released (Figure 1C; Supplementary Video 1), as well as smelly gas; the latter consists of alkanes and alkenes (6). During the balloon angioplasty procedure, the balloon (Pacific™ Plus PTA Catheter, 3.0 mm × 80 mm × 130 cm; Medtronic, Minneapolis, MN, USA) is fully inflated at 8 atm, and the pressure is quickly released after being maintained for 3 min. The laser catheter, balloon, and guidewire were advanced and withdrawn gently. No signs of perforation of plaques were noted.

### Micro-CT and Carotid Artery CTA Images

Samples were scanned with a SkyScan1176 system [hardware version A, software version 1.1 (build 11)] using the following settings: 45 kV, 556 μA, and 8.70 μm pixel size, with a 0.2-mm aluminum filter and a round scanning trajectory. Since the maximum detection length of this system is 3 cm, each sample was scanned in 3-cm sections. Scanning of the specimens was done without a 360° rotation around the vertical axis and a single rotation step of 0.3°. All images were reconstructed by using the software NRecon (version 1.6.10.4) with the reconstruction engine GPUReconServer (version 1.6.10), provided with the corresponding micro-CT scanner system SkyScan1176 (version 1. 1). Reconstruction was done with 8.70 μm pixel size. Three-dimensional (3D) reconstruction of both the micro-CT and carotid artery CT angiography (CTA) images was performed separately on the following two software: CTAn (version 1.17.7.2) and Mimics Medical (version 21.0; Materialise, Leuven, Belgium). Artery/plaque lumen was set as a region of interest and then reconstructed. Subsequently, stl type files generated from CTAn or Mimics Medical were imported into 3-matic Medical (version 13.0; Materialise, Leuven, Belgium) for fine-tuning: smoothens the surface and flattens single-point spikes of the 3D models.

### Histology

The collected saline was smeared on a glass slide by means of a blood smear. The slides were placed into a constant temperature oven set at 37°C overnight, and then hematoxylin–eosin (HE) staining and Movat's pentachrome staining were performed. Five pictures were randomly taken at × 400 magnification for further analysis. The pictures were imported into Fiji (version 1.52u) and the area and Feret diameter data of the debris were obtained through the “Analyze Particles” function. Device-treated plaques were embedded in paraffin and then sectioned serially with a thickness of 5 μm. Since, clinically, the stenosis degree of the narrowest lumen was one of the surgical indications, 10 slices used for HE staining were randomly selected from the 1-cm-long plaque containing the narrowest lumen. Masson trichrome staining and Movat's pentachrome staining were then performed on the next serial slices. After being dehydrated with increasing concentrations of ethanol and cleared through two changes of xylene, the slides were mounted on neural resin followed by a glass coverslip. The slides were observed and photographed using a light microscope. Histological and quantitative analyses were executed with Fiji (version 1.52u) and R (4.0.2).

### Pathological Classification of Carotid Atherosclerotic Plaque

For the pathological classification of the carotid atherosclerotic plaque, the standards established by the American Heart Association (AHA) are generally used. However, this study has a contradiction between determining the pathological type of the carotid atherosclerotic plaque and reducing the volume of the plaque. The carotid atherosclerotic plaque wax and slices after the pathological classification of the AHA did not meet the requirements of the volume reduction experiment. All patients with carotid atherosclerosis and stenosis did not undergo carotid MRI before CEA. Therefore, this study can only perform a simple classification of the plaque samples used in the experiments through the HE staining results of the reduced carotid atherosclerotic plaque. The classification and corresponding judgments are based on the following: lipid plaque—the ratio of the total lipid core to the area of the plaque is ≥0.4 or the distance between the lipid core and the plaque lumen is <200 μm, then the total area standard is relaxed to 30%; calcified plaque—the ratio of the total calcification to the area of the plaque is ≥0.4 or the calcified nodule is <200 μm from the plaque lumen, then the total area standard is relaxed to 30%; fibrous plaque—after the above, two types of plaques are eliminated and are classified as fibrous plaques (7–9).

### SEM Observation

The surface and edge of the working area left after treatment were observed with a scanning electron microscope (SEM) in order to compare the microscopic morphology of the lumen surface after treatment. The treated plaque samples from three groups were pre-fixed for 2 h with 2.5% glutaraldehyde fixative solution and then rinsed overnight with 0.1 M phosphate buffer solution. The samples were post-fixed with 1% osmium tetroxide for 2 h and dehydrated in a series of graded ethanol solutions. After being washed with 3-methylbutylacetate and then dried to the critical point (VFD-21S t-Butanol Drying Device; Vacuum Device Inc., Mito, Japan), the specimens were attached to an aluminum mount with an adhesive tape (Carbon Adhesive Tape, SPI Supplies Division of STRUCTURE PROBE, West Chester, PA, USA). The specimens were then observed with SEM (Quanta250FEG, Thermo Fisher Scientific Inc., Waltham, MA, USA) at 20 kV.

### Statistical Analysis

Statistical analysis was performed using SPSS 26.0 software (IBM, Armonk, NY, USA). The Kolmogorov–Smirnov and Shapiro–Wilk normality tests were used to test whether the data conformed to a normal distribution or not. The mean ± standard deviation or the mean ± standard error was used to represent measurement data with a normal distribution; the median (quartile) was used for measurement data with a non-normal distribution. Use rate represents the classification data. Under the premise of normal distribution and uniform variance, a two-sample independent *t*-test was used to compare the values of the two groups of measurement data; one-way ANOVA was used for multigroup comparisons. If these two premises were not met, a non-parametric test was used for comparison. GraphPad Prism 8.3.0 (GraphPad Software, Inc., San Diego, CA, USA) and RStudio (version 1.3.1073) were used for the diagrams. A value of ^*^p < 0.05, ^**^p < 0.01, or ^***^p < 0.001 was considered statistically significant.

## Results

### Study Population

In total, 18 patients (14 men and 4 women) with an average age of 67.3 ± 8.28 years had undergone CEA, and 18 carotid plaques were harvested with an average length of 3.47 ± 0.95 cm. The demographic data are shown in Table 1.

**Table 1:** Baseline data of carotid artery stenosis patients.

**Characteristics**	**Data**
Mean plaque length (cm)	3.47 ± 0.95
Age (years)	67.3 ± 8.28
Gender (male, %)	66.7
BMI (kg/m^2^)	22.91 ± 3.05
SBP (mmHg)	147.8 ± 20.0
DBP (mmHg)	79.8 ± 12.4
Ipsilateral stenosis (%)	78.8 ± 9.2
Symptomatic (%)	44.0
Ischemic heart disease (%)	0
PAD (%)	5.6
Diabetes (%)	38.9
Hypertension (%)	77.8
Hyperlipidemia (%)	27.8
Smoking (%)	27.8
Alcohol usage (%)	27.8
Aspirin (%)	66.7
Statin (%)	72.2
Antihypertensive drugs (%)	77.8
Hypoglycemic drugs (%)	38.9
Triglyceride levels	3.23 ± 7.23
Cholesterol levels	3.87 ± 0.98
Low-density lipoprotein levels	2.07 ± 0.72
High-density lipoprotein levels	1.16 ± 0.21
Blood glucose (mmol/L)	6.21 ± 1.72
HbA1c (mmol/L)	6.56 ± 1.06

*Continuous data are presented as the mean ± standard deviation. Categorical data are given as a percentage*.

*BMI, body mass index; SBP, systolic blood pressure; DBP, diastolic blood pressure; PAD, peripheral artery disease; HbA1c, glycated hemoglobin A1c*.

### Microdebris

**Figure 2:**
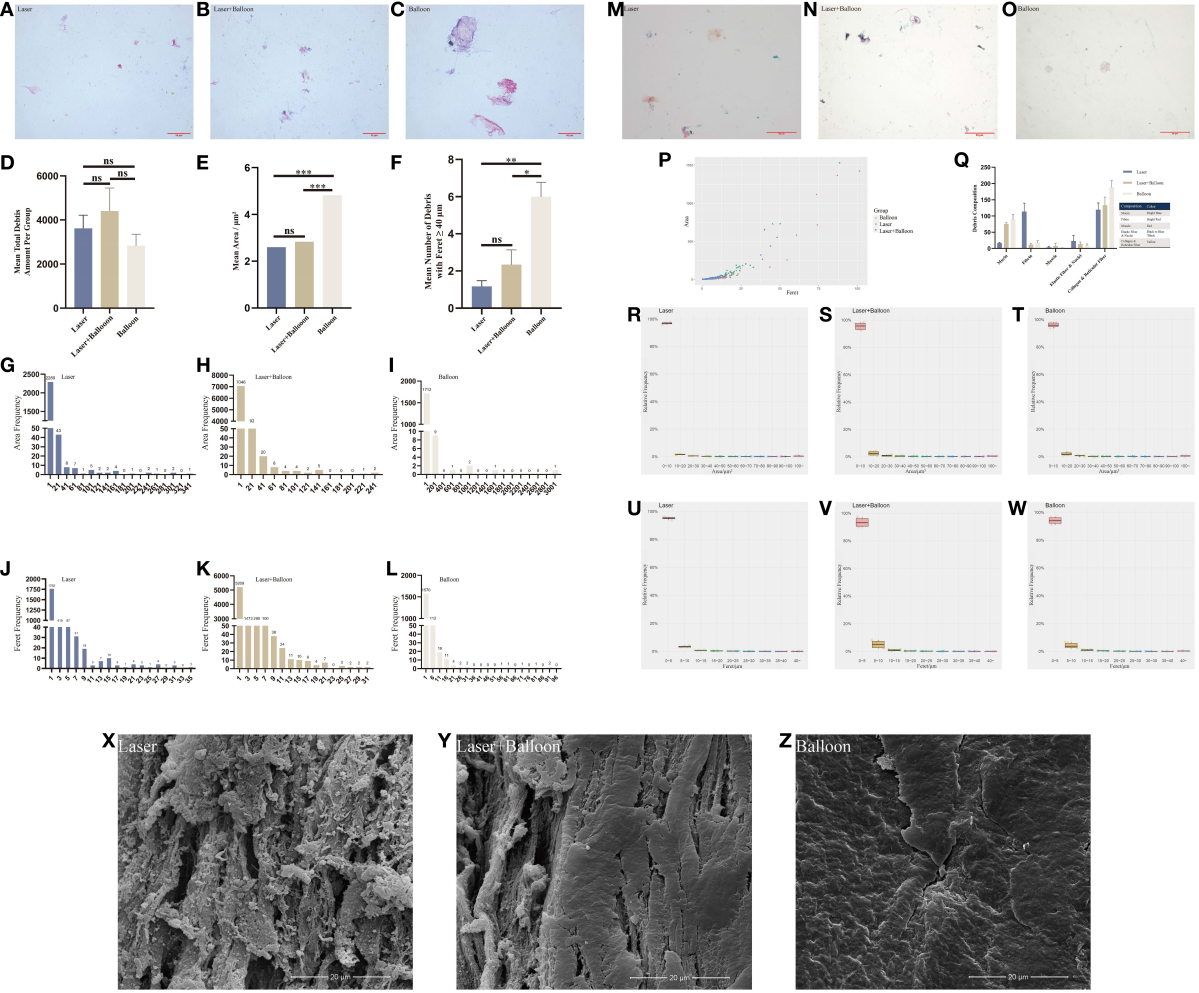
Analysis of debris and observation of the plaque lumen surface after device treatments. **(A–C)** Hematoxylin–eosin staining images of the microdebris from the three groups. **(D–F)** Comparison of the mean total debris amount **(D)**, mean area **(E)**, and mean number of debris with Feret ≥ 40 μm **(F)** of the three groups. **(G–L)** Histogram of the microdebris area and Feret of three carotid plaques from the three groups. **(M–O)** Movat's pentachrome staining images of microdebris from the three groups. **(Q)** Debris composition of the three groups based on the quantification of Movat's pentachrome staining. **(P)** Scatter plot of the area and Feret of three samples from the three groups. **(R–W)** Relative frequency map of the debris area and Feret of the three groups. **(X–Z)** Micromorphological images of the plaque lumen surface. Laser, excimer laser alone group; Laser + Balloon, excimer laser combined with balloon group; Balloon, balloon alone group; Feret, maximum caliper diameter; ns, not significant. *p < 0.05, **p < 0.01, ***p < 0.001.

Histopathological staining indicated the presence of microdebris in all collected saline (Figures 2A–C). After counting, we found that the mean total amount of microdebris of group 3 was higher than those of the other two groups (Figure 2D; Table 2). However, no significant difference was found among the three groups. The area, the maximum caliper diameter (Feret), and the minimum caliper diameter (MinFeret) were analyzed. Data of the above parameters for each group are shown in Table 2. One sample was selected from each group, and the area and Feret distribution of each sample are exhibited in Figures 2G–L. The MinFeret distribution of the three samples can be found in Supplementary Figures A–C. The mean microdebris area of group 2 was significantly bigger than those of the other two groups (Figure 2E). Figures 2R–T exhibited that more than 90% of the debris area data of all groups were concentrated in the interval (0, 10) μm^2^, while the Feret data of all groups were concentrated in the interval (0, 5) μm (Figures 2U–W). The Minferet data of all groups can be found in Supplementary Figures F–H. However, there was no difference among groups when comparing the mean Feret and the mean MinFeret values (Supplementary Figures D,E). Clinically, distal embolic protection devices are widely used in CAS procedures, with pore sizes ranging from 40 to 200 μm (Table 3). Therefore, we compared the mean number of debris with Feret ≥ 40 μm among the groups. As with the mean area, we found that group 2 has significantly more debris with Feret ≥ 40 μm; no difference was noted between the laser and laser + balloon groups (Figure 2F). The scatter plot of the area and Feret of debris from the three groups suggested that the majority of the debris consisted of relatively smaller ones. In addition, debris from group 2 has a wider range of Feret distribution (Figure 2P). To identify the composition of the microdebris, Movat's pentachrome staining was performed (Figures 2M–O). As shown in Figure 2Q, the debris composition varied from group to group. The major components of the three groups were collagen and reticular fiber; muscle was the minor one. Besides, mucin and fibrin were the two components that were quite different among groups.

**Table 2:** Quantitative data of the total debris and lumen of three groups.

**Group**	**Laser**	**Laser + balloon**	**Balloon**
**Debris**			
Total amount	21,666	26,402	16,968
Mean amount	3,611 ± 1,475.4	4,400 ± 2,567.9	2,828 ± 1,266.7
Mean area (μm^2^)	2.60 ± 17.53	2.83 ± 22.25	4.81 ± 69.03
Mean Feret (μm)	1.96 ± 2.43	1.98 ± 2.60	2.02 ± 4.14
Mean MinFeret (μm)	1.15 ± 1.42	1.18 ± 1.50	1.23 ± 2.42
Total number of Feret ≥ 40 μm	7.00	14.0	36.0
Mean number of Feret ≥ 40 μm	1.17 ± 0.75	2.33 ± 1.97	6.00 ± 1.90
**Lumen**			
Mean area of treated plaques (mm^2^)	9.32 ± 11.80	7.09 ± 5.56	8.37 ± 5.55
Mean Feret of treated plaques (mm)	4.90 ± 2.51	6.83 ± 4.65	5.44 ± 2.42
Mean MinFeret of treated plaques (mm)	2.99 ± 1.92	3.11 ± 1.80	2.63 ± 1.21
Mean area enlargement (mm^2^)	4.05 ± 3.20	5.40 ± 4.51	3.77 ± 2.55
Mean Feret enlargement (mm)	2.51 ± 1.71	4.33 ± 3.31	1.54 ± 0.75
Mean MinFeret enlargement (mm)	1.68 ± 1.10	2.11 ± 1.52	0.88 ± 0.62

*Continuous data are presented as the mean ± standard deviation*.

*Feret, maximum caliper diameter; MinFeret, minimum caliper diameter*.

**Table 3:** Distal embolic protection devices.

**Distal protection device**	**Pore size (μm)**
BSX Filter-wire EZ™	110
Cordis Angioguard^®^ XP	100
ev3 Spider FX™	80–160
Invatec Fibernet	>40
Abbott RX Accunet	115
Abbott Emboshield NAV^6™^	120
GORE^®^ Embolic Filter	100

*Data were from the Internet*.

### Lumen Gain of Debulked Plaque

**Figure 3:**
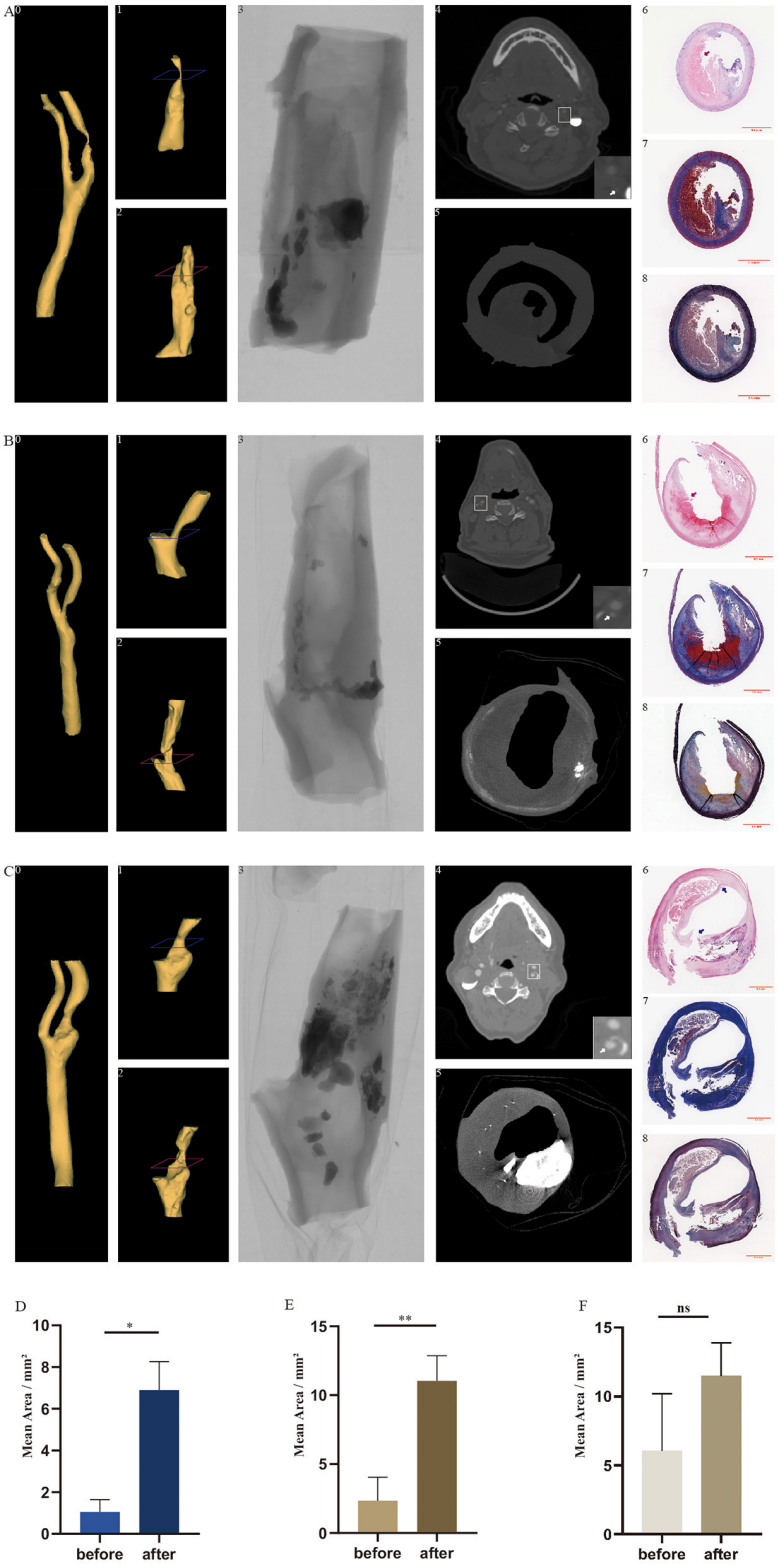
*In vivo* carotid arteries and *ex vivo* treated plaques with co-registration between CTA, Micro-CT, 3D reconstruction, and histology in longitudinal and transversal views. Three-dimensional reconstruction of the lumen of carotid arteries (*A0, B0*, and *C0*) and treated plaques (*A2, B2*, and *C2*) based on images from CTA and Micro-CT. The reconstructed lumen of *in vivo* plaque before the experiment (*A1, B1*, and *C1*) was cropped from the reconstructed arteries. Perspective view of the plaque taken by Micro-CT (*A3, B3*, and *C3*). The cross-sections of CTA (*A4, B4*, and *C4*) and Micro-CT (*A5, B5*, and *C5*) correspond to the levels demonstrated in A1, B1, and C1 and in A2, B2, and C2 (*blue* and *red*). *White arrow* indicates the internal carotid artery. Hematoxylin–eosin staining (*A6, B6*, and *C6*), Masson trichrome staining (*A7, B7*, and *C7*), and Movat's pentachrome staining (*A8, B8*, and *C8*) correspond to the cross-sections of Micro-CT. *Red arrows* indicate the curved boundaries of the plaque lumen after excimer laser debulking. *Blue arrows* indicate the cracks produced by balloon angioplasty. CTA, computed tomography angiography; Micro CT, micro-computed tomography; 3D reconstruction, three-dimensional construction. *p < 0.05, **p < 0.01. **(A)** CTA, Micro-CT, and histological images of sample from group 1. **(B)** CTA, Micro-CT, and histological images of sample from group 3. **(C)** CTA, Micro-CT, and histological images of sample from group 2. **(D)** Mean lumen area of plaques in group 1 before and after treatment. **(E)** Mean lumen area of plaques in group 3 before and after treatment. **(F)** Mean lumen area of plaques in group 2 before and after treatment.

The selection of each plaque used was based on the plaque stenosis obtained from pre-surgical examinations, including computer tomography, Doppler ultrasound, or digital subtraction angiography. After the excimer laser and balloon treatments, the carotid plaques were embedded in paraffin, sectioned, and underwent HE, Masson trichrome, and Movat's pentachrome staining. There was no evidence of thermal necrosis found on the edge of the debulking region after laser ablation (Figure 3, A6 and B6). Cracks were noted after the plaques were squeezed by a ballo on (Figure 3, C6). The area, Feret, and MinFeret values of the lumen were calculated and analyzed. By using Fiji software, lumen data were obtained from 10 randomly selected slices from above and below the location of the narrowest lumen. The average lumen areas of the treated plaques of the three groups were 9.32, 7.09, and 8.38 mm^2^, respectively. Lumen data of the plaques before treatments were obtained from presurgical CTA images using the Mimics Medical software. The lumen area of the carotid atherosclerotic plaques in groups 1 and 3 changed significantly before and after debulking, while that of group 2 did not change significantly (Figures 3D–F). By comparing the lumen areas pre- and post-procedure, we noted that group 3 had the highest lumen area enlargement (Table 2). In summary, after the *ex vivo* procedure, the stenosis of plaques in each group decreased variously. Additionally, stenosis reduction in group 3 was more than that of the other two groups.

### Pathological Types of Plaques Used in Each Group

Based on clinical application and our postoperative pathological classification, carotid atherosclerotic plaque was classified as fibrous plaque, lipid plaque, and calcified plaque. The corresponding numbers of plaque types used in groups 1 and 3 were 2, 4, and 0, respectively; in group 2, however, these were 3, 1, and 2, respectively.

### Scanning Electron Microscopy

The lumen surface morphology of the carotid plaques produced by the laser, balloon, or laser + balloon can be seen in Figures 2X–Z. Fibers underneath the plaque endothelium were exposed after laser ablation, while balloon squeezing caused crakes on the lumen surface. Additionally, fibers and cracks also occurred in group 3.

### 3D Reconstruction

Three-dimensional models of the plaque lumen after treatments (3D-after; Figure 3, A2, B2, and C2) and the artery lumen (3D-artery; Figure 3, A0, B0, and C0) were reconstructed from the original micro-CT images and clinical CTA images, respectively. Based on the position and the length of the 3D-after models, we cropped the corresponding 3D-artery models and obtained 3D models of the plaque lumen before treatments (3D-before; Figure 3, A1, B1, and C1). Unlike the 3D-before models, the figures of the 3D-after models are more irregular (Figures 3A–C). The cross-sections of the CTA (Figure 3, A4, B4, and C4), micro-CT (Figure 3, A5, B5, and C5), and histology (Figure 3, A6–A8, B6–B8, and C6–C8) are demonstrated at two levels in Figure 3 (A1–A2, B1–B2, and C1–C2, blue and red). Plaque burden was decreased as the plaque composition was removed after excimer laser debulking, according to the histological images in Figure 3.

## Discussion

CAS is considered a valuable alternative to CEA for high-risk patients. Although the plaque cannot be removed like during CEA, plaque remodeling after CAS is of great importance to the prognosis. Stent-related complications include stent thrombosis and in-stent restenosis, which can be predicated by insufficient stent expansion (10–15). During the CAS procedure, adequate stent expansion means greater luminal gain. Factors leading to a suboptimal stent expansion include the severity of calcification, vessel morphology, and plaque load (16–19). In the vessel preparation period, plaque remodeling strategies to dilate the calcified lesions and plaque modification facilitate the entrance of the distal filter and the adequacy of stent expansion. Strategies to modify carotid plaques could be theoretically achieved by debulking carotid plaques. Hoping to achieve a CEA-like outcome by using endovascular intervention equipment, we named it Endo-CEA and set our sights on peripheral debulking atherectomy devices. According to the type of atherectomy performed and the basic technique features, peripheral debulking devices can be categorized into the following four main types: directional atherectomy (e.g., SilverHawk, ev3 Inc., Plymouth, MN, USA) rotational atherectomy (e.g., JetStream, Pathway Medical Technologies, Inc., Redmond, WA, USA) orbital atherectomy (e.g., DiamondBack 360°, Cardiovascular Systems Inc., Saint Paul, MN, USA) and photoablation atherectomy (e.g., Spectranetics CVX-300, Philips, San Diego, CA, USA) (20, 21). By comparing the features as well as the accessibility of each mentioned device (22), we finally focused on the excimer laser system. To our knowledge, there was no previous research applying an excimer laser as a debulking device for human carotid plaques.

In the experiment, we used an 8-F excimer laser catheter with a diameter of 2.5 mm. The excimer laser manufacturer's recommendation for the selection of laser catheters is as follows: the ratio of the diameter of the laser catheter to the diameter of the target blood vessel should be 1:1.5. From this, inversely, the minimum vessel diameter matched by a 2.5-mm laser catheter must be at least 3.75 mm. In addition, the operating instructions of the excimer laser system (CVX-300) used in the experiments clearly stated that the recommended blood vessel diameter corresponding to a 2.5-mm laser catheter is 3.8 mm or more. Based on this, we chose a 3.0-mm balloon as the comparison object. In order to compare differences between the excimer laser and the balloon in blood vessel preparation, we set the balloon expansion pressure at 8 atm to simulate pre-expansion. The velocity of laser catheter advancement was conducted as per the manufacturer's specification. Catheter advancement was repeated five times, as we found that the laser debulking efficiency after repeating three times was not satisfactory in a preliminary experiment. We found no thermal damages on the sharp cutting edges after excimer laser ablation (Figure 3, A6 and B6), which is in conformity with previous studies (23, 24). According to clinical guidelines, patients received surgical or interventional treatment based on a judgment of carotid artery stenosis and symptoms (25, 26). In the clinical setting, residual stenosis is an important indicator for evaluating the effect of carotid artery revascularization. In our study, to simplify calculations, plaque lumen enlargement was used as the main measurement to describe efficiency, other than residual stenosis. The results showed that an excimer laser and balloon could accomplish lumen enlargement to various degrees, and the combination of both devices yielded the maximum lumen area gain. In addition, excimer laser ablation showed higher efficiency than did balloon angioplasty. Excimer laser debulking enlarges the plaque lumen by removing the plaque composition, as shown in Figure 3, while balloon angioplasty displaces the plaque components. Plaque displacement was not permanent. The fiber components in the plaque, especially the elastic fiber, retracted after the inflation pressure had been removed. This might explain why plaque lumen enlargement in group 2 was less efficient than that in the other two groups. After 3D reconstruction of the micro-CT images, we noticed that the shape of the lumen models was irregular. The reasons for these irregular shapes were the curved boundaries left after excimer laser debulking (Figure 3, A6 and B6, red arrow) and the uneven thickness of the plaque sample (Figure 3, A6, B6, and C6). The thinner area is prone to deformation. The plaque samples lost attachment to the artery wall after being removed from human bodies, leading to a loss of support to maintain an approximately circular shape. On the contrary, the samples were locally concave or convex, especially the thinner part. The sample slices and micro-CT images exhibited the laser ablation region located in the tissue area, other than the calcification nodules. The boundary created by laser ablation was distributed along the edge of the calcification nodules, and no significant defect was found in the nodules. Although an excimer laser is capable of removing calcium, as the operative instruction indicated, our results suggest that excimer laser debulking running at parameters of 45 mJ/mm^2^ and 25 Hz might not be suitable for highly calcified lesions. Therefore, we assumed that removal of the calcification nodules could be achieved by boosting the energy density, increasing the frequency, and repeating more operations; meanwhile, a higher lumen enlargement would be obtained.

In current practice, various cerebral embolic protection devices are used to avoid intraoperative stroke during CAS. Among the devices, distal filters are the most widely used. The function of a distal filter is to capture the microembolus, which requires the pore size to be equal to or smaller than the size of the embolus. We summarized the information of several protection devices and found the pore size to range from 40 to 200 μm (Table 3). However, our debris data showed that debris with Feret ≥ 40 μm made up quite a small proportion of the total amount. This meant that clinically used protection devices were unable to completely prevent the microemboli from flowing into the brain with blood, causing symptomatic or a silent ischemic stroke. Since more than 95% of the debris Feret was smaller than 40 μm and could not be captured by embolic protection devices, the incidence of those complications would probably be high. In reality, the perioperative stroke incidence is relatively low (1, 27, 28). This phenomenon might be explained by the findings of Carson et al., which revealed that the microemboli causing cerebral microvessel occlusion were translocated outside the vessel lumen, realizing recanalization (29). The diameters of the fibrin clots, cholesterol emboli, and the polystyrene microspheres used in their study were in ranges of 8–20 and 10–15 μm. This protective mechanism suggested that debris of these sizes might not lead to observable detrimental complications. An embolus ranging in size from 60 to 500 μm was noted to cause ischemic stroke and cognitive decline (30–32). While the pathological processes and the outcomes of debris of other sizes remain unknown. Besides, after carotid artery angioplasty and stenting with distal protection devices were applied, the ischemic area was noticed throughout the brain and was predominantly located in the deeper layers of the cortex in the distribution of the middle cerebral artery (33). In addition, filters with high capture efficiency are associated with favorable clinical outcomes (34). Therefore, we hypothesized that debris of unresearched sizes was responsible for the prejudicial complications and that new cerebral protection devices should cover these sizes. But the “slow flow” phenomenon was found to be associated with the design of distal protection devices (35–37). Therefore, when upgrading distal protection devices, a balance between the filter parameters, such as the pore size, pore number, vascular resistance, and blood flow patency, must be cautiously controlled. Besides, the dilemma between capture capacity and the “slow flow” phenomenon might suggest that the currently widely used cerebral protective devices, represented by the distal filter, are probably not suitable for this new type of debulking therapy. We then sorted out the composition of the collected debris with Movat's pentachrome staining. The differences in the debris composition of the three groups were concentrated in mucin and fibrin. We did not list out the calcified particles, which does not mean that no calcified components were noted. This is because the Movat's pentachrome stain we used to detect debris components was not designed to detect calcified particles. Theoretically, an excimer laser can break down the carotid plaque tissue and transform it into H_2_O and gas (photochemical effect). However, not all molecules receive the energy that the excimer laser releases. In addition, the photomechanical and photothermal effects play a role in the formation of microdebris. That is to say, microdebris are “stripped” from the carotid plaque, without the physical and chemical properties being affected substantially. Therefore, the debris components are associated with the plaque composition, i.e., the histopathological type. In this study, the discovery of the debris composition has a certain coincidence. In further research, increasing the sample size of plaques with different pathological types might reveal some regularities.

The present study did not include other debulking devices such as SilverHawk, JetStream, and DiamondBack 360°. It is because these devices are not accessible in our region. Besides, the working process such as the rotation and/or vibration of these devices is considered too irritating for the carotid artery to sustain. The carotid sinus is located at the beginning of the internal carotid artery and functions as a sensitive baroreceptor. Undoubtedly, stimulation to the carotid sinus could possibly result in a severe drop in heart rate and blood pressure. Too intense and too much irritation is life-threatening.

Recently, an emerging technique—transcarotid artery revascularization (TCAR)—has been under heated discussion. During the TCAR procedure, reverse blood flow was established out of the blood pressure difference between patients' common carotid artery and femoral vein. This design gave us a flash of inspiration, which is achieving a CEA-like outcome, i.e., maximally removing the carotid plaque endovascularly by combining the use of an excimer laser, balloon, and a reversal flow technique. However, the pore size of the outside filter of the ENROUTE Neuroprotection System is 200 μm, which is considered less efficient based on our results.

## Limitations

The number of plaque samples used in the study is relatively small. Studies with larger samples are needed to confirm our findings. Furthermore, applying devices on carotid plaques classified by pathology and imaging and finding out the specific plaque types with the highest debulking efficiency could guide precise clinical application in the future. Besides, the most suitable and efficient functioning parameters ought to be discovered. In our study, the velocity of the saline used to wash out and collect debris was much slower than the blood flow rate *in vivo*. Therefore, the total amount of debris is definitely smaller than the actual amount. In the future, experiments that more closely resemble *in vivo* situations need to be conducted. Additionally, studies on the combination of an excimer laser, balloon, and reverse blood flow technique should be designed and performed to verify the original conjecture of “Endo-CEA.”

## Conclusion

The present study was conducted to investigate the safety and effectiveness of applying an excimer laser in debulking human carotid atherosclerotic plaques. Also, we compared the excimer laser to a widely used balloon and explored the efficiency of their combination. Our macroscopic and microscopic results suggest that the excimer laser + balloon presented the highest efficiency. Besides, we proposed a transformable hypothesis that might provide a new option for the treatment of carotid stenosis.

## Data Availability Statement

The original contributions presented in the study are included in the article/Supplementary Material, further inquiries can be directed to the corresponding author/s.

## Ethics Statement

The studies involving human participants were reviewed and approved by Ethics Committee of Shanghai Pudong Hospital. The patients/participants provided their written informed consent to participate in this study.

## Author Contributions

BY, WS, JT, and KY participated in the design of the study and drafted the manuscript and contributed to the clinical data analysis and were involved in revising the manuscript. JT, YD, and KY conducted the experiments and participated in the data collection and analysis. All authors have read and approved the final manuscript.

## Funding

This research was supported by The Outstanding Clinical Discipline Project of Shanghai Pudong New Area (Grant No. PWYgy-2018–08), the Shanghai Municipal Natural Science Foundation (Grant No. 18ZR1433900), and the Program for Medical Key Department of Shanghai (Grant No. ZK2019A10).

## Conflict of Interest

The authors declare that the research was conducted in the absence of any commercial or financial relationships that could be construed as a potential conflict of interest.

## Publisher's Note

All claims expressed in this article are solely those of the authors and do not necessarily represent those of their affiliated organizations, or those of the publisher, the editors and the reviewers. Any product that may be evaluated in this article, or claim that may be made by its manufacturer, is not guaranteed or endorsed by the publisher.
