# Supplementary figures and images for: Endovascular Debulking of Human Carotid Plaques by Using an Excimer Laser Combined With Balloon Angioplasty: An ex vivo Study
Source: Front Cardiovasc Med. 2021 Sep 20;8:700497. doi: 10.3389/fcvm.2021.700497 (PMC8488149; doi:10.3389/fcvm.2021.700497)

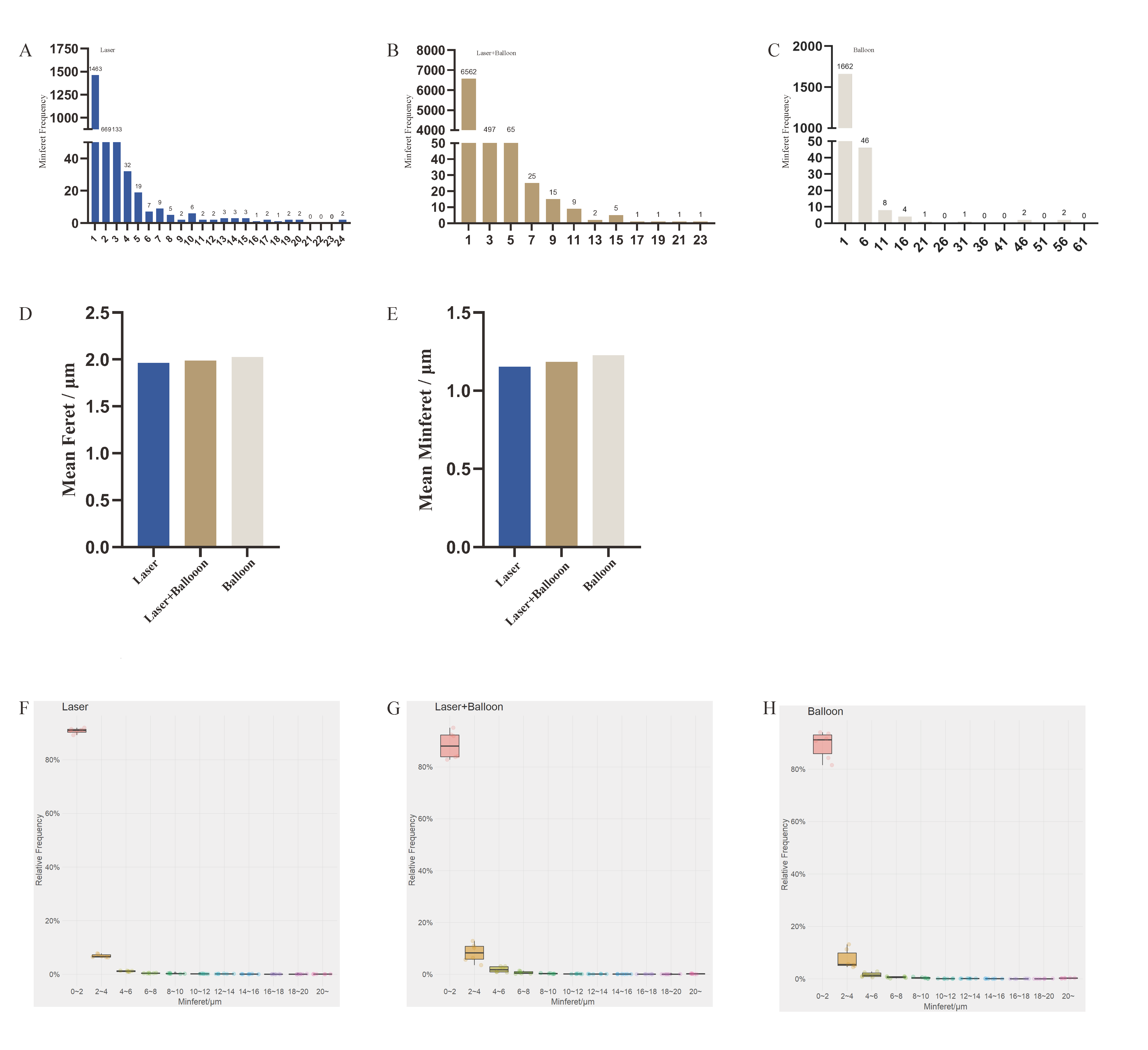

Supplement: Supplementary file 2 [file Image_1.TIF]
